# Spectrum of Ischemic Heart Disease Throughout a Woman’s Life Cycle

**DOI:** 10.14797/mdcvj.1331

**Published:** 2024-03-14

**Authors:** Smitha Narayana Gowda, Sai sita Garapati, Karla Kurrelmeyer

**Affiliations:** 1 Methodist DeBakey Cardiology Associates; 2 Houston Methodist Hospital, Houston, Texas, US

**Keywords:** MINOCA, INOCA, vasospasm, coronary microvascular dysfunction, SCAD, ischemic heart disease in women

## Abstract

Ischemic heart disease (IHD) is the leading cause of morbidity and mortality in both genders; however, young women fare the worst, likely reflecting the more complex spectrum of IHD in women when compared to men. Substantial sex-based differences exist in the underlying risk factors, risk enhancers, presentation, diagnosis, and pathophysiology of IHD that are mainly attributed to the influence of female sex hormones. This article reviews the spectrum of IHD including obstructive epicardial coronary artery disease (CAD), myocardial infarction with no obstructive coronary artery disease, ischemia with no obstructive coronary artery disease, spontaneous coronary artery dissection, coronary microvascular dysfunction, vasospastic angina, and coronary thrombosis/embolism that occur in women throughout various stages of their life cycle. We aim to update clinicians on the diagnosis and management of these various types of IHD and highlight where further randomized controlled studies are needed to determine optimal treatment and inform guideline-directed medical therapy.

## Introduction

Cardiovascular disease (CVD) is the leading cause of morbidity and mortality in women worldwide, with most deaths and disability being attributed to ischemic heart disease.^1^ Particularly concerning is the rising number of deaths in the United States since 2010, especially given the significant decline in the prior decade.^2^ Most of this increase was due to deaths in younger women ages 35 to 54 years old,^2^ with some studies quoting a 2-fold higher mortality in younger women compared to younger men.^3^ Over 400,000 women in the United States died from CVD in 2019, with ischemic heart disease (IHD) being implicated in more than half of those deaths.^1^ Given the complex landscape of IHD in women, this review will discuss both obstructive and nonobstructive acute and chronic conditions, including atherosclerotic obstructive epicardial coronary artery disease (CAD), ischemia with no obstructive coronary artery disease (INOCA), myocardial infarction with no obstructive coronary artery disease, spontaneous coronary artery dissection, coronary microvascular dysfunction (CMD), vasospasm, coronary thrombosis, and embolism. In addition, we will delve into how hormonal changes, risk factors, risk enhancers, age, and stress impact these various forms of IHD in women and discuss current diagnosis and treatment recommendations.

## Premenopausal and Pregnant Women

### Spontaneous Coronary Artery Dissection

Spontaneous coronary artery dissection (SCAD) is a non-iatrogenic dissection of the epicardial coronary artery resulting from the development of an intramural hematoma between the coronary artery layers (intima, media, adventitia), causing ischemia and myocardial infarction (MI).^4^ It occurs independent of trauma or atherosclerosis and has been associated with variations in hormone levels during menstruation, pregnancy, and while taking hormonal therapy for contraception, infertility, and replacement therapy.^5,6^ It is an important cause of acute MI in women < 50 years old, accounting for up to 35% of acute coronary syndrome (ACS) cases in this demographic.^7,8^ It is also the main culprit in pregnancy-associated MI, accounting for 14.5% to 43% of all MIs in these patients.^9,10^ Although it can occur any time during the peripartum period, > 70% occur postpartum, usually within the first week.^9^ Patients with pregnancy-related SCAD are more likely to present with shock, left main disease, and multivessel dissections compared to nonpregnant patients with SCAD.^9^ There is a higher prevalence of pregnancy-related SCAD in women with multigravida and women who used infertility treatments, developed pre-eclampsia, and had advanced maternal age.^9^

SCAD has also been associated with arteriopathies including Marfan syndrome, Loeys-Dietz, Ehlers Danlos in 5% to 9% of patients,^11^ and fibromuscular dysplasia (FMD) in > 50%.^12,13,14^ While genetic testing may be considered, experts recommend arterial imaging from the brain to pelvis, usually with computed tomography angiography (CTA) or magnetic resonance angiography to exclude FMD in other vascular beds.^15^ Patients with SCAD are more than twice as likely to report extreme physical or emotional stress prior to the onset of SCAD compared to patients presenting with non-SCAD MI.^16^ Epidemiology studies demonstrate that 87% to 95% of all SCAD cases occur in middle-aged women (mean age 44 to 53) with significantly less CV risk factors than age and sex-matched cohorts, however, SCAD has been reported in teenage women to nonagenarians.^17,18^

**Figure 1:**
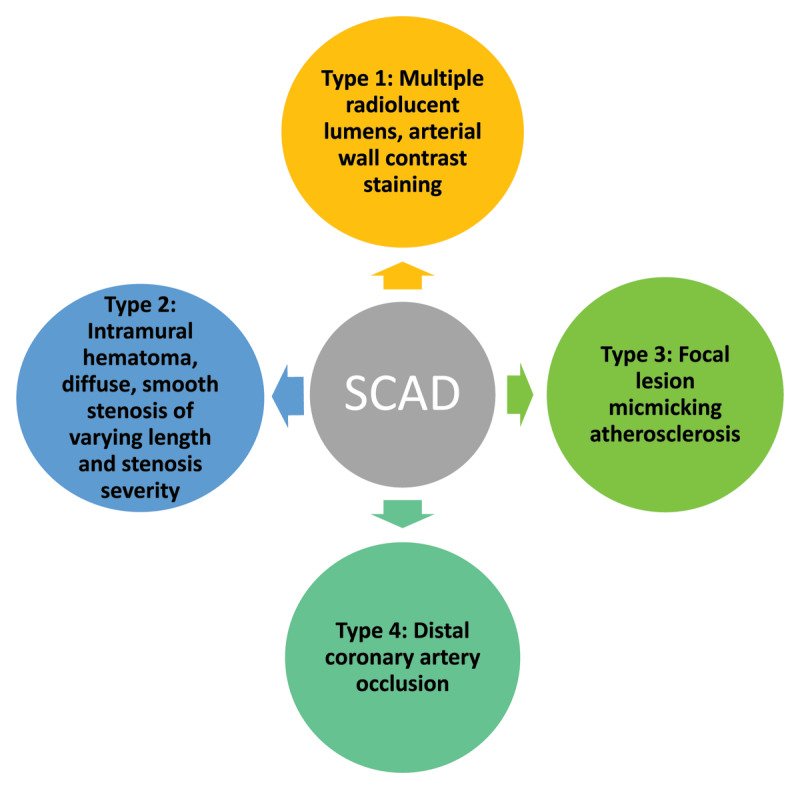
Yip-Saw angiographic classification for spontaneous coronary artery dissection (SCAD).

**Figure 2:**
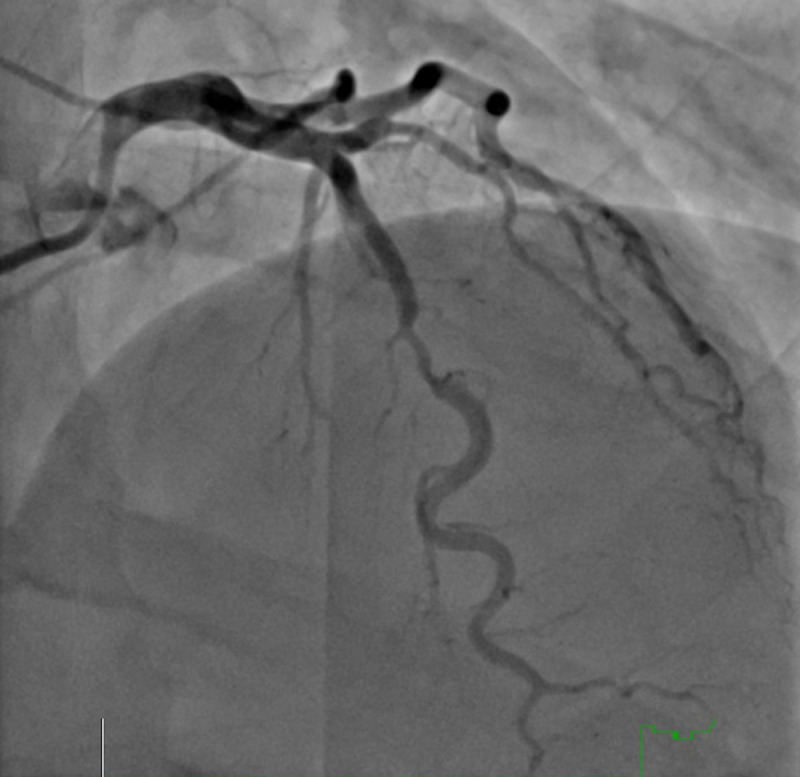
Invasive coronary angiogram of type 3 spontaneous coronary artery dissection (SCAD) in a 54-year-old woman presenting with non-ST-elevation myocardial infarction (NSTEMI). Type 3 SCAD is the most difficult type to distinguish from obstructive coronary artery disease.

The diagnosis of SCAD is challenging and requires a high degree of suspicion. The Yip-Saw classification scheme based on angiographic appearance is helpful in diagnosing SCAD, but even the most experienced angiographers find it difficult to distinguish SCAD from epicardial obstructive CAD (Figures 1, 2).^19,20^ SCAD most commonly affects the mid to distal coronary artery, with the left anterior descending coronary artery being affected in most cases.^19,20^ Intracoronary nitroglycerine and intracoronary imaging with optical coherence tomography (OCT) and intravascular ultrasound (IVUS) can help distinguish SCAD from a ruptured plaque by direct visualization of the intramural hematoma and/or dissection flap, with OCT providing the greatest yield due to higher spatial resolution.^4^ Once the diagnosis is made, one of the most difficult decisions for an interventional cardiologist to make is to not intervene. Percutaneous coronary intervention (PCI) should be avoided unless the patient is clinically unstable presenting with ST-elevation MI (STEMI), ventricular arrhythmias, cardiogenic shock, or sudden cardiac arrest given the heightened risk for propagation of the intramural hematoma, iatrogenic coronary artery dissection, and abrupt vessel occlusion during coronary intervention.^15^ Computed tomography angiography (CTA) is a useful noninvasive modality recommended for diagnosis in stable patients and as a surveillance tool to assess for subsequent coronary artery healing. It is especially helpful in diagnosing proximal SCAD and the presence of FMD in other vascular beds but has limitations in diagnosing SCAD in both small and distal vessels.^21,22^

Unlike ACS due to obstructive coronary atherosclerosis, medical management is recommended for stable SCAD patients. Remarkably, healing of SCAD lesions occurs in most patients (84.3-97%) with medical management.^23,24^ There are no randomized controlled trials (RCTs) to guide medical management of SCAD. However, experts recommend that SCAD patients receive the same guideline-directed medical therapy as ACS patients—including nitrates, beta blockers, aspirin, P2Y_12_-receptor inhibitor, statin, heparin, and morphine as needed for pain relief in the acute setting.^25^ Anticoagulation should be discontinued as soon as a diagnosis of SCAD is made to prevent possible intramural hematoma extension.^15^ Long-term statin therapy should be determined based on primary prevention guidelines until informed by RCTs such as SAFER-SCAD (Statin and Angiotensin-converting Enzyme Inhibitor on Symptoms in Patients with SCAD).^19,26^ There is no consensus on dual antiplatelet therapy (DAPT) in patients with SCAD managed conservatively due to concern for intramural hematoma extension and bleeding complications that come with long-term use of DAPT. Most experts recommend long-term low-dose aspirin therapy after a short duration of DAPT.^26^ Given the ambiguity concerning the duration of antiplatelet use and use of beta blockers in these patients, there is an ongoing RCT evaluating short- versus long-term dual antiplatelet treatment and the benefit of beta blockers in SCAD patients.^27^

Revascularization with percutaneous coronary intervention or coronary artery bypass graft surgery (CAB) may still be indicated in a minority of patients with hemodynamic instability, life-threatening arrhythmia, or left main dissection and/or involvement of the proximal epicardial vessels.^1^ CAB has been historically reserved for those patients in whom PCI has failed or who have on-going ischemia with left main dissection. Early mortality after CAB in a small cohort of 20 patients was determined to be 5%; however, no additional mortality after hospital discharge was observed at 5 years, and the 5-year event rate in those receiving CAB was similar to those treated medically.^12^

The recurrence rate in SCAD varies between 10% and 30% across studies, with multiple associated features and triggers including hypertension, emotional and physical stressors, coronary tortuosity, migraine headache, FMD, and other arteriopathies.^19^ After multivariate modeling that controlled for age at first SCAD, year of first SCAD, and history of FMD, pregnancy did not increase the risk of recurrent SCAD in women with a history of previous SCAD.^28^ A prospective observational SCAD cohort study that followed 327 SCAD patients, 90% women, and 62.7% with fibromuscular dysplasia for a median follow-up of 3.1 years found that 10% had recurrent SCAD. Multivariate analysis demonstrated that hypertension increased the incidence of recurrent SCAD by more than 2-fold, and beta-blocker use was associated with a 64% reduction in recurrent SCAD.^19^

### Obstructive Epicardial Atherosclerotic CAD in High-Risk Premenopausal Women

Mortality due to CAD, has decreased for most groups over the years, but not for younger women.^29,30^ Data from the US VIRGO (Variation in Recovery: Role of Gender on Outcomes of Young Acute Myocardial Infarction Patients) registry demonstrated that obstructive epicardial atherosclerotic CAD is the most prevalent form of IHD in women between 18 and 55 years old, accounting for 86.3% of all acute myocardial infarctions (AMI).^31^ Younger women with obstructive epicardial atherosclerotic CAD have more comorbidities, including diabetes mellitus, chronic obstructive pulmonary disease, chronic kidney disease, obesity, anemia, peripheral vascular disease (PAD), and autoimmune diseases.^32,33^ However, diabetes portends the worst prognosis. Diabetes has been shown to increase the risk of death more than 6-fold when compared with nondiabetic women. Women with diabetes also have an increased risk of death and cardiovascular events compared with diabetic men, with one study demonstrating a 40% excess risk.^34^ Delayed presentation and diagnosis plus treatment differences between women and men presenting with ACS have also been associated with excess mortality in younger women. Several studies have reported that women present up to an hour later with their symptoms, are less likely to be correctly diagnosed, less likely to receive invasive coronary angiography and revascularization, less likely to be discharged on secondary prevention meds, and less likely to be referred to cardiac rehabilitation.^35,36,37,38,39^

### Coronary Artery Thrombosis/Embolization

Coronary artery thrombosis/embolization is a rare cause of myocardial infarction in young women with nonobstructed coronary arteries (MINOCA). In the VIRGO registry, just 2 women out of 2,690 participants were diagnosed with coronary artery embolization, with one having a history of familial thrombophilia, specifically factor V Leiden, which has been shown to increase risk by 1.7- to 3.7- fold.^33^ However, in a recent case series, it was found to be the second most frequent cause of ACS during pregnancy, occurring in up to 30% of cases. It is hypothesized to occur secondary to alterations in the coagulation and fibrinolytic systems, resulting in a 4-fold increased risk of venous thrombosis during pregnancy, putting pregnant women with peripartum cardiomyopathy, atrial fibrillation, mechanical prosthetic valves, and atrial septal defects including patent foramen ovale particularly at risk.^40^

## Perimenopausal Woman

### Myocardial Infarction with No Obstructive Coronary Arteries

MINOCA is an umbrella term used to describe all patients presenting with symptoms or electrocardiographic changes suggestive of ACS and troponin elevation but are found to have nonobstructive CAD, defined as < 50% stenosis of a major epicardial coronary artery on invasive coronary angiography. The definition of MINOCA has varied in the literature, with some studies narrowing the criteria to coronary etiologies including coronary thrombosis, coronary embolism, coronary microvascular dysfunction, coronary spasm, myocardial bridging, plaque erosion/rupture, and MINOCA.^41^ Other studies include coronary etiologies in addition to noncoronary cardiac causes—including cardiac trauma, cardiomyopathy, cardiotoxins, myocarditis, strenuous exercise, Takotsubo cardiomyopathy, and transplant rejection—as well as noncardiac causes—including acute respiratory distress syndrome, allergic/hypersensitivity reactions, end-stage renal failure, inflammation, pulmonary embolism, sepsis, and stroke.^25^ Approximately 1% to 15% of women with ACS referred for invasive coronary angiography are diagnosed with MINOCA.^41^ More than one-third of these women have plaque rupture or ulceration by IVUS.^42,43^

Data from the VIRGO registry that restricted the criteria for MINOCA to coronary etiologies demonstrated that MINOCA patients are younger, have less hypertension, diabetes, dyslipidemia, smoking, obesity, family history of CAD, prior AMI, or PAD when compared to patients with AMI due to obstructed coronary arteries. They also are less likely to present with STEMI and less likely to undergo revascularization. In addition, discharge management varied, with MINOCA patients less likely to receive aspirin, beta blockers, angiotensin converting enzyme inhibitors, angiotensin receptor blockers, or statins on discharge and less likely to be referred to cardiac rehabilitation. Women were five times more likely to present with MINOCA than men (14.9% versus 3.5%, OR 4.84; 95% CI, 3.29-7.13) and both sexes presented with chest pain more than 85% of the time. MINOCA was not a benign diagnosis in the VIRGO registry since those diagnosed with MINOCA were just as likely as those with AMI due to obstructed coronary arteries to present with cardiac arrest, reduced ejection fraction, and heart failure.^33^ Various studies have shown that MINOCA patients have lower mortality at 1 year than patients with AMI due to obstructive CAD; however, it is still 3.2% to 4.5%,^44^ significantly higher than age- and sex-matched healthy cohorts.

The 2023 European Society of Cardiology (ESC) guidelines for the management of acute coronary syndrome recommend that if MINOCA is diagnosed following invasive coronary angiography, functional assessment is needed, including left ventriculography, left ventricular end-diastolic pressure, measurement of coronary microvascular function, and coronary vasoreactivity. If functional coronary angiography does not establish a cause for MINOCA, then noninvasive imaging with echocardiography, computed tomography (CT), and/or cardiovascular magnetic resonance imaging (CMR) is recommended if clinically indicated.^25^ CMR has been shown to identify the underlying etiology in up to 87% of patients and is recommended prior to discharge to increase its diagnostic yield.^45^

Treatment should be tailored to the final diagnosis to decrease morbidity and mortality. The SWEDEHEART study evaluated 9,136 MINOCA patients with a median follow-up of 4 years and found a significant reduction in major adverse cardiac events (MACE) with statins, angiotensin-converting enzyme inhibitors (ACEIs)/angiotensin receptor blockers (ARBs), and a trend for improved outcomes with beta blockers, but no significant benefit with DAPT at 1 year.^46^

### Ischemia with No Obstructive Coronary Arteries

Ischemia with no obstructive coronary arteries (INOCA) describes patients with symptoms suggestive of ischemia and frequently positive noninvasive functional stress tests but are found to have nonobstructive CAD defined as < 50% diameter reduction or fractional flow reserve (FFR) ≥ 0.8 of a major epicardial coronary artery on invasive coronary angiography (ICA).^47^ Greater than 50% of patients undergoing coronary angiography for stable angina have nonobstructive epicardial coronary arteries with a higher prevalence observed in women (60-70%) than in men (30%).^48^ Multiple large registries including the Women’s Ischemic Syndrome Evaluation (WISE) have demonstrated that patients with INOCA have an increased risk for death, MI, rehospitalization, and repeat ICA compared to those with normal coronaries but less than those with obstructive CAD. On multivariate analysis, diabetes, older age, hypertension, and smoking predicted worse outcomes.^48,49,50,51^ The CONFIRM registry (Coronary CT Angiography Evaluation for Clinical Outcomes: An International Multicenter Registry) enrolled 23,854 subjects with angina but without known CAD and showed that all-cause mortality was similar between women and men with INOCA and with those with obstructive 1-vessel CAD. However, older women fared worse than older men and had a 5-fold higher risk of all-cause mortality than younger women.^52^

INOCA can be caused by coronary microvascular dysfunction (CMD) or coronary vasospasm or both. The 2021 American Heart Association (AHA)/American College of Cardiology (ACC) Chest Pain Guidelines recommend either invasive coronary function testing, stress positron emission tomography, or stress CMR with myocardial blood flow reserve (MBFR) in patients with INOCA depending on local availability and expertise, with a preference for an invasive assessment due to its higher diagnostic yield attributed to its ability to diagnose coronary vasospasm (IIa, level of evidence B).^53^ This recommendation is based on the CorMicA (Coronary Microvascular Angina) trial, an RCT that enrolled patients suspected of having INOCA to undergo coronary angiography and invasive coronary physiology testing including FFR, coronary flow reserve (CFR), and index of microvascular resistance (IMR) followed by vasoreactivity testing with acetylcholine. Study participants were 70% female, 50% had CMD, 17% had vasospastic angina (VSA), and 20% had both CMD and VSA. Participants were randomized to intervention and control arms. The control arm received standard of care, while the intervention arm received appropriate medical therapy based on the following final diagnosis obtained from invasive testing: MVA, VSA, mixed MVA/VSA, obstructive CAD, or noncardiac causes. There was no difference in MACE between the two arms, but there was a significant reduction in angina and improvement in quality of life at 6 months and 1 year of follow-up in the intervention arm.^54^

### Coronary Microvascular Dysfunction

The coronary microcirculation is responsible for resistance circuits in the heart and is instrumental for coronary blood flow regulation. At rest, myocardial oxygen extraction is at maximum capacity, thereby necessitating augmented coronary blood flow to meet any escalated demand for oxygen. Coronary microvascular dysfunction (CMD) is present when there is decreased ability of the microvasculature to vasodilate and increase coronary blood flow during stress, causing supply-demand mismatch and ischemia. The pathophysiology implicated in CMD includes endothelial dysfunction, smooth muscle cell dysfunction, microvascular remodeling, extramural compression, autonomic dysfunction, and inflammation. Under normal conditions, the endothelium releases vasoactive substances including nitric oxide, leading to vasodilation in response to stimuli such as acetylcholine and physiological triggers (eg, exercise). With endothelial dysfunction, the pharmacological and physiological stimuli lead to decreased vasodilatory response or even vasoconstriction, leading to decreased blood flow. Smooth muscle dysfunction is evidenced by a decreased vasodilatory response to adenosine, regadenoson, dipyridamole, or papaverine, particularly observed in patients with comorbidities such as diabetes, hypertension, metabolic syndrome, obesity, chronic kidney disease, and heart failure with preserved ejection function. CMD has been classified into four main types based on coexisting clinical syndromes: (1) CMD in the absence of myocardial diseases and obstructive CAD; (2) CMD in myocardial diseases; (3) CMD in obstructive CAD; and (4) iatrogenic CMD.^55,56,57,58,59,60,61,62^

**Figure 3:**
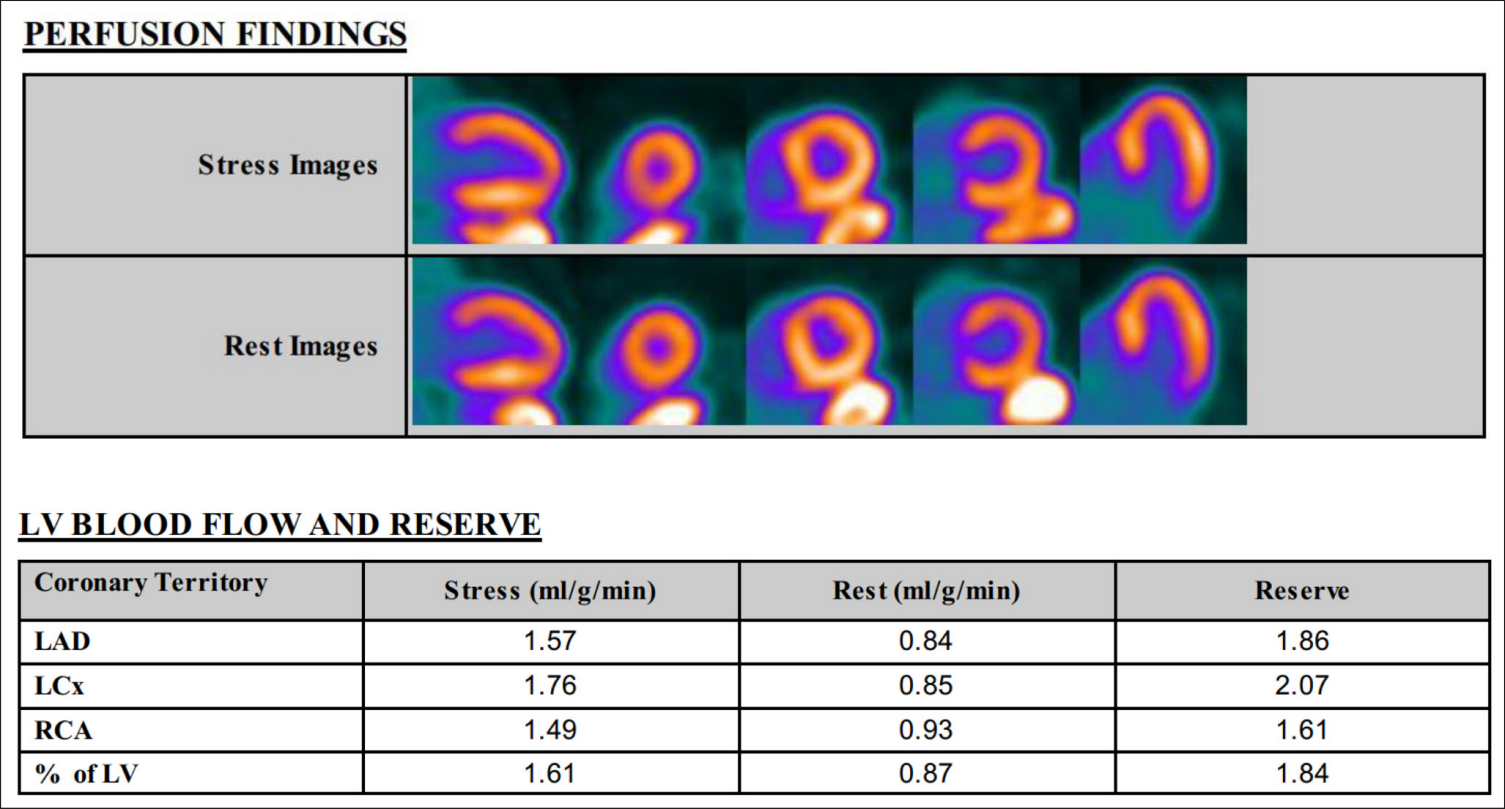
Positron emission tomography with myocardial flow reserve (MFR) showing normal perfusion with impaired MFR of 1.84 (normal MFR > 2) in a 76-year-old woman with microvascular angina. LV: left ventricular; LAD: left anterior descending artery; LCx: left circumflex artery; RCA: right coronary artery

Patients with CMD typically present with microvascular angina (MVA), which resembles angina in those with obstructive CAD, with symptoms including rest or exertional angina, exertional dyspnea, and decreased exercise tolerance. Notably, conventional diagnostic modalities for IHD, including exercise ECG, single-photon emission computed tomography (SPECT), and echocardiography have low diagnostic power for detecting CMD. During exercise ECG testing, patients may exhibit ischemic changes on the ECG along with chest pain, while SPECT may reveal reversible perfusion defects; however, regional wall motion abnormalities are rarely demonstrated. The CMD Coronary Vasomotor Disorders International Study (COVADIS) established the diagnostic criteria for CMD, which requires MVA, evidence of ischemia by noninvasive testing, no functionally significant epicardial coronary artery obstruction by either ICA or CTA, and evidence of CMD by invasive coronary function testing or stress PET or stress CMR with MBFR. These criteria were incorporated into the AHA/ACC/ESC guidelines for the management of patients with chronic coronary artery disease (Table 1, Figure 3).^63,64,65,66,67^

**Table 1:** American Heart Association, American College of Cardiology, and European Society of Cardiology guidelines for the management of patients with chronic coronary artery disease. COVADIS: Coronary Vasomotor Disorders International Study Group; CMD: coronary microvascular disease; CAD: coronary artery disease; FFR: fractional flow reserve; CTA: computed tomography angiography; ECG: electrocardiography; IMR: microcirculatory resistance; TIMI: Thrombolysis in Myocardial Infarction


COVADIS DIAGNOSTIC CRITERIA FOR CMD

**Symptoms of myocardial ischemia**

Effort and/or rest angina Angina equivalents (ie, shortness of breath)

**Absence of obstructive CAD** (< 50% diameter reduction or FFR > 0.80) by

Coronary CTA Invasive coronary angiography

**Objective evidence of myocardial ischemia**

Ischemic ECG changes during an episode of chest pain Stress-induced chest pain and/or ischemic ECG changes in the presence or absence of transient/reversible abnormal myocardial perfusion and/or wall motion abnormality

**Evidence of impaired coronary microvascular function**

Impaired coronary flow reserve (cut-off values depending on methodology use between ≤ 2.0 and ≤ 2.5) Coronary microvascular spasm, defined as reproduction of symptoms, ischemic ECG shifts but no epicardial spasm during acetylcholine testing Abnormal coronary microvascular resistance indices (eg, IMR > 25) Coronary slow flow phenomenon, defined as TIMI frame count > 25


Coronary microvascular function can be determined by measuring CFR or microcirculatory resistance (IMR). CFR can be measured noninvasively using stress PET or stress CMR with MBFR, or transthoracic Doppler echocardiography to measure left anterior descending coronary artery flow. CFR is a ratio of hyperemic to resting myocardial blood flow. Hyperemia is induced using regadenoson or adenosine. PET myocardial blood flow is most commonly used and provides robust and reproducible assessment of myocardial flow reserve (MFR). An MFR < 2 has been shown to be a strong predictor of cardiovascular mortality and major adverse cardiovascular outcomes.^68,69,70^ Invasive coronary function testing can be used to assess CFR and index of IMR and has an added value of assessing microvascular spasm with acetylcholine provocation testing. CFR can be obtained invasively using either the thermodilution method or Doppler peak flow velocity method at rest and hyperemia induced by adenosine infusion. A CFR of < 2 or 2.5 is associated with microvascular dysfunction and adverse outcomes. IMR at hyperemia is defined as hyperemic mean distal pressure multiplied by mean transit time, with > 25 demonstrated to be abnormal. IMR ≥ 25 and CFR < 2 was associated with worse MACE, but isolated increased IMR is not correlated with worse outcomes.^71^

Management of CMD is challenging but was informed by the CorMicA trial, which demonstrated improvement in symptoms with smoking cessation, healthy lifestyle changes, aspirin, statin, ACEI, as-needed sublingual nitroglycerin, first-line antianginal treatment with beta blocker, second-line antianginal treatment with non-dihydropyridine calcium channel blocker if beta blockers are ineffective or not tolerated, and third-line add-in therapy with dihydropyridine calcium channel blocker, nicorandil, or ranolazine for persistent symptoms.^54^ The Women’s Ischemia trial to Reduce Events in Non-Obstructive CAD (WARRIOR) is an ongoing multicenter RCT evaluating whether maximally tolerated statin, ACEIs or ARBs, and low-dose aspirin will reduce MACE in women with INOCA and should offer more insight into optimal medical therapy for women with CMD and other forms of INOCA.^71^

### Vasospastic Angina

Vasospastic angina (VSA) is caused by either focal or diffuse vasoconstriction of a single or multiple epicardial coronary arteries or of the microvasculature. The exact prevalence of VSA is hard to determine since diagnosis frequently requires documentation of coronary spasm during provocation testing with acetycholine or ergonovine during ICA, which is rarely done due to safety concerns even though it has been shown to be safe, with just 3.2% of patients developing ventricular tachycardia/ventricular fibrillation and 2.7% developing bradyarrhythmias. A meta-analysis of INOCA patients showed that 16% to 73% have VSA, with a higher prevalence in Asian than Western populations. Epicardial coronary vasospasm is more common in men, while microvascular spasm is more common in women.^54,72^ Patients with VSA are younger, with fewer cardiac risk factors than patients with obstructive CAD. Smoking is a major risk factor for VSA. Other identified triggers for VSA include cocaine use, cold exposure, hyperventilation, emotional stress, and alcohol consumption. The pathophysiology of VSA is thought to be multifactorial, including vascular smooth muscle hyperreactivity, endothelial dysfunction, autonomic nervous system dysfunction, oxidative stress, and genetic predisposition.^72^

The diagnosis of VSA requires angina with ischemic ST-segment changes on ECG. Because these symptoms are transient and occur mainly at rest, with most episodes occurring from midnight to the early morning hours, diagnosis can be challenging. Ambulatory ECG monitoring may be helpful. A definitive diagnosis is imperative since VSA can rarely present with STEMI, arrythmias, syncope and sudden cardiac death. ICA with provocation testing is considered positive when it causes angina, ischemic ECG changes, and > 90% reduction in epicardial coronary artery diameter either with contrast injection, catheter manipulation, or following intravenous acetylcholine or ergonovine. The development of angina and ischemic ST-segment changes without epicardial coronary spasm suggests microvascular spasm.^73^

Calcium channel blockers and long-acting nitrates along with controlling triggers and risk factors, especially smoking cessation, are recommended. Overall prognosis of INOCA patients with VSA is favorable if high-risk clinical features such as STEMI, arrythmias, syncope, and sudden cardiac death are not present.^54,67,74^

## Postmenopausal Woman

### Obstructive Epicardial Atherosclerotic CAD in Postmenopausal Women

Registries have shown that women with ACS due to obstructive epicardial CAD are postmenopausal, with a mean age of 60 to 70 years, up to 10 years older than men. One-third are diabetic, which frequently coexists with hypertension and chronic kidney disease. A third are frail, a quarter are ≥ 75 years and severely frail, with female sex predicting frailty and frailty predicting death despite appropriate treatment for ACS. Postmenopausal women are more likely to have single-vessel disease when presenting with STEMI but multivessel disease when presenting with NSTEMI.^75,76^ Women have more bleeding, likely secondary to non-weight-adjustment of anticoagulants, although bleeding significantly improved with use of radial access for ICA and PCI. After adjustment for age and comorbidities, there was no significant difference in mortality and MACE between postmenopausal women and men presenting with ACS.^77^

In chronic coronary disease, data from the CONFIRM registry using coronary computed tomography angiography (CCTA) on 24,950 patients showed that women tend to develop coronary atherosclerosis 12 years later than men and have less plaque burden and more nonobstructive disease. However, postmenopausal women with the highest atherosclerotic burden had the highest risk of MACE, with hypertension predicting MACE in women and hypercholesterolemia in men.^78^ These differences are hypothesized to be due to the effects of endogenous estrogen. Estrogen has been shown to protect against endothelial dysfunction possibly through upregulation of nitric oxide and downregulation of angiotensin 1 receptors and angiotensin-converting enzyme protecting against renin-angiotensin system induced vasoconstriction. Therefore, loss of estrogen has been associated with hypertension and endothelial dysfunction manifested as INOCA, MINOCA, CMD, or VSA. Loss of estrogen has also been associated with a more atherogenic lipid profile, with increases in low density lipoprotein, total cholesterol, lipoprotein a, and a decline in high density lipoprotein as well as increases in central adiposity resulting in an increased risk of insulin resistance, metabolic syndrome, and diabetes accelerating the atherosclerotic process.^79^ However, this study from the CONFIRM registry demonstrates that women have the most to gain from CCTA since prior studies have shown that the identification of plaque on CCTA leads to intensification of medical therapy and up to a 40% reduction in MI and death.^78,80^

## Conclusions

**Figure 4:**
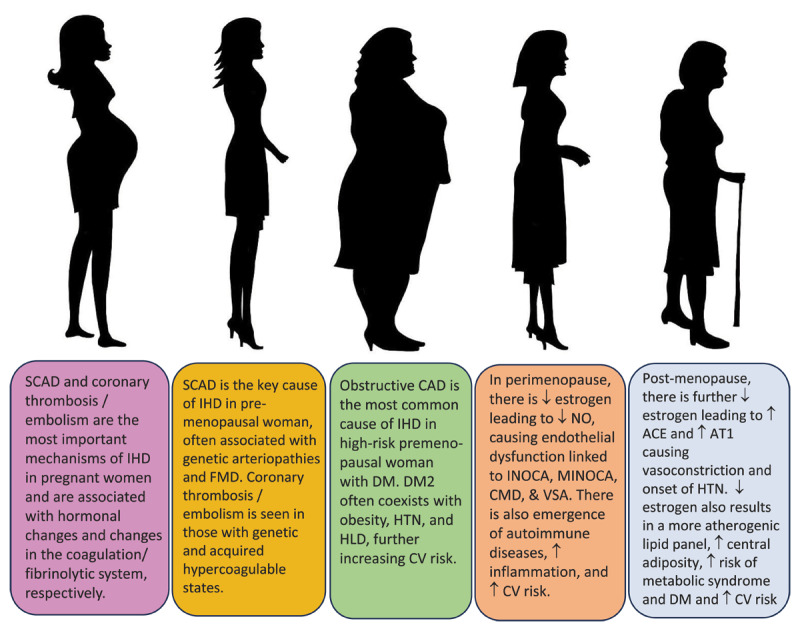
Spectrum of ischemic heart disease (IHD) in women. SCAD: spontaneous coronary artery dissection; FMD: fibromuscular dysplasia; DM2: diabetes mellitus type 2; CAD: coronary artery disease; CV: cardiovascular; CMD: coronary microvascular dysfunction; INOCA: ischemia with no obstructive coronary arteries; MINOCA: myocardial infarction with no obstructive coronary arteries; VSA: vasospastic angina; ACE: angiotensin converting enzyme; AT1: angiotensin 1 receptor; HTN: hypertension

This review highlights the spectrum and complexity of IHD in women (Figure 4). It describes emerging concepts on how hormonal changes, risk factors, risk enhancers, age, and stress impact the various forms of IHD as well as sex differences in presentation, diagnosis, and treatment, illustrating the importance of awareness, education, and research in improving outcomes, especially in younger women who are at highest risk of death. Further RCTs are needed to determine optimal treatment and inform guideline-directed medical therapy, particularly for the nonobstructive coronary syndromes, for this goal to be realized.

## Key Points

- Spontaneous coronary artery dissection is an important cause of acute myocardial infarction in women under 50 years of age, particularly in the context of pregnancy. Pregnancy-related spontaneous coronary artery dissection (SCAD) exhibits a propensity for involvement of the left main coronary artery, multivessel disease, and a higher likelihood of presenting with cardiogenic shock compared with non-pregnancy-related SCAD.
- Approximately 1% to 15% of women with acute coronary syndrome are diagnosed with myocardial infarction with no obstructed coronary arteries (MINOCA). Women are five times more likely than men to present with MINOCA, and cardiac magnetic resonance imaging has been shown to identify the underlying pathology in 87% of patients with an unidentifiable cause on functional coronary angiography.
- Half of patients undergoing angiogram for stable angina are diagnosed with ischemia with no obstructive coronary artery disease (INOCA), with a higher prevalence among women than men. These patients have been shown to have similar one year mortality as patients with obstructive single-vessel coronary artery disease.
- Women develop obstructive coronary artery disease 10 years later than men, owing to the protective effects of endogenous estrogen. However, postmenopausal women with the highest plaque burden have the highest risk of major adverse cardiovascular events.
